# An EBC/Plasma miRNA Signature Discriminates Lung Adenocarcinomas From Pleural Mesothelioma and Healthy Controls

**DOI:** 10.3389/fonc.2021.643280

**Published:** 2021-06-15

**Authors:** Alice Faversani, Chiara Favero, Laura Dioni, Angela Cecilia Pesatori, Valentina Bollati, Matteo Montoli, Valeria Musso, Andrea Terrasi, Nicola Fusco, Mario Nosotti, Valentina Vaira, Alessandro Palleschi

**Affiliations:** ^1^ Division of Pathology, Fondazione IRCCS Ca’ Granda—Ospedale Maggiore Policlinico, Milan, Italy; ^2^ EPIGET Lab, Department of Clinical Sciences and Community Health, University of Milan, Milan, Italy; ^3^ Department of Preventive Medicine, Fondazione IRCCS Ca’ Granda—Ospedale Maggiore Policlinico, Milan, Italy; ^4^ Division of Thoracic Surgery and Lung Transplantation, Fondazione IRCCS Ca’ Granda Ospedale Maggiore Policlinico, Milan, Italy; ^5^ Division of Molecular Biology, Biomedical Center, Faculty of Medicine, LMU Munich, Martinsried, Germany; ^6^ Department of Biomedical, Surgical and Dental Sciences, University of Milan, Milan, Italy; ^7^ Department of Pathophysiology and Transplantation, University of Milan, Milan, Italy

**Keywords:** microRNA, lung cancer, malignant pleural mesothelioma, exhaled breath condensate, liquid biopsy, volatile biopsy

## Abstract

**Background:**

Despite significant improvement in screening programs for cancers of the respiratory district, especially in at-risk subjects, early disease detection is still a major issue. In this scenario, new molecular and non-invasive biomarkers are needed to improve early disease diagnosis.

**Methods:**

We profiled the miRNome in exhaled breath condensate (EBC) and plasma samples from fourteen patients affected by lung AdCa, nine healthy subjects. miRNA signatures were then analyzed in another neoplasia of the respiratory district, i.e. pleural mesothelioma (n = 23) and subjects previously exposed to asbestos were used as controls for this cohort (n = 19). Selected miRNAs were analyzed in purified pulmonary neoplastic or normal epithelial and stromal cell subpopulation from AdCa patients. Finally, the plasmatic miRNA signature was analyzed in a publicly available cohort of NSCLC patients for data validation and *in silico* analysis was performed with predicted miRNA targets using the multiMiR tool and STRING database.

**Results:**

miR-597-5p and miR-1260a are significantly over-expressed in EBC from lung AdCa and are associated with AdCa. Similarly, miR-1260a is also up-regulated in the plasma of AdCa patients together with miR-518f-3p and correlates with presence of lung cancer, whereas let-7f-5p is under-expressed. Analysis of these circulating miRNAs in pleural mesothelioma cases confirmed that up-regulation of miR-518f-3p, -597-5p and -1260a, is specific for lung AdCa. Lastly, quantification of the miRNAs in laser-assisted microdissected lung tissues revealed that miR-518f-3p, 597-5p and miR-1260a are predominantly expressed in tumor epithelial cells. Validation analysis confirmed miR-518f-3p as a possible circulating biomarker of NSCLC. *In silico* analysis of the potentially modulated biological processes by these three miRNAs, shows that tumor bioenergetics are the most affected pathways.

**Conclusions:**

Overall, our data suggest a 3-miRNAs signature as a non-invasive and accurate biomarker of lung AdCa. This approach could supplement the current screening approaches for early lung cancer diagnosis.

## Introduction

Neoplasia of the respiratory district is among the leading cause of tumor related death worldwide. Among those are lung cancer and pleural mesothelioma, two diseases whose risk is further increased by environmental and occupational exposure to carcinogens, such as asbestos (1–3). Screening programs for such neoplasia are currently based on low-dose computed tomography (LDCT). The NELSON trial showed that the use of LDCT for lung cancer screening reduced lung cancer mortality of about 26% in men and 39–61% in women (4). Nevertheless, shared guidelines for nodules assessment and interpretation are still to be optimized to reduce the false-positive rate, limit the number of invasive procedures for benign disease and to avoid overtreatment of precancerous lesions (1, 3). Lastly, LDCT screening has important economical clues for National Health systems, and a selection of patients who might benefit more by LDCT is warranted (3).

Among lung cancers, the lung adenocarcinoma histotype represents the most frequent type of Non-Small Cell Lung Cancer (NSCLC) and it is characterized by a poor prognosis. In particular, patients affected by NSCLC have a 5-year predicted survival expectancy of 15.9% and a high recurrence rate (5). Despite advances in the field of tailored medical therapy in metastatic disease, an approach in the early stages is still the cornerstone of lung cancer treatment. Therefore, early diagnosis remains the most effective approach to detect the disease at an earlier, asymptomatic, and potentially curable stage. Similarly, although risk factors of MPM are well known, the current standard for MPM diagnosis relies on pleural biopsies.

In this scenario, the addition of non-invasive biomarkers to thoracic cancer screening protocols could reduce overtreatment rate and improve the eligibility selection for LDCT screening, overall reducing patients stress and sanitary costs. Further, biomarkers could support the clinical algorithm also in discriminating among different cancer types (3, 6). More efforts are needed to identify new molecular biomarkers to complement early NSCLC and MPM diagnosis by non-invasive techniques.

microRNAs (miRNAs) are small non coding RNAs which regulate the translation of their targets mRNAs. miRNAs are involved in numerous physiological and pathological cellular processes (7); in particular, miRNAs participate in cancer progression with an oncogenic or tumor suppressor role (7). Further, miRNAs can be detected in normal and tumor tissues, but also in biological fluids such as serum and plasma (8), through their secretion *via* exosomes or as Ago2 protein complexes (9), where they can act as extracellular messengers. In this context, different studies showed the importance of miRNAs as circulating biomarkers in lung cancer and their use for liquid biopsy (10, 11).

Previously, we showed that two extracellular vesicles (EV)-associated plasmatic miRNAs distinguish patients with malignant pleural mesothelioma from cancer-free subjects previously exposed to asbestos (12) and that avatar mice of NSCLC retain tissue-specific and circulating miRNAs signatures of the primary tumor (13). Recently, it has been suggested that exhaled breath condensate (EBC)-miRNAs, the so-called volatile biopsy, could represent novel, non-invasive reliable biomarkers of respiratory diseases (14, 15), including lung cancer (16).

In this study, we aimed to identify miRNA signatures useful for lung cancer screening using minimally invasive procedures, such as EBC and plasma collection. To this end, we analyzed the EV-associated miRNome in EBC and plasma derived both from patients affected by NSCLC and healthy control subjects. Furthermore, we compared these signatures to the ones associated with malignant pleural mesothelioma to provide preliminary clues about the potential of circulating miRNA as a tool for diseases discrimination.

## Materials and Methods

### Patients

**Figure 1:**
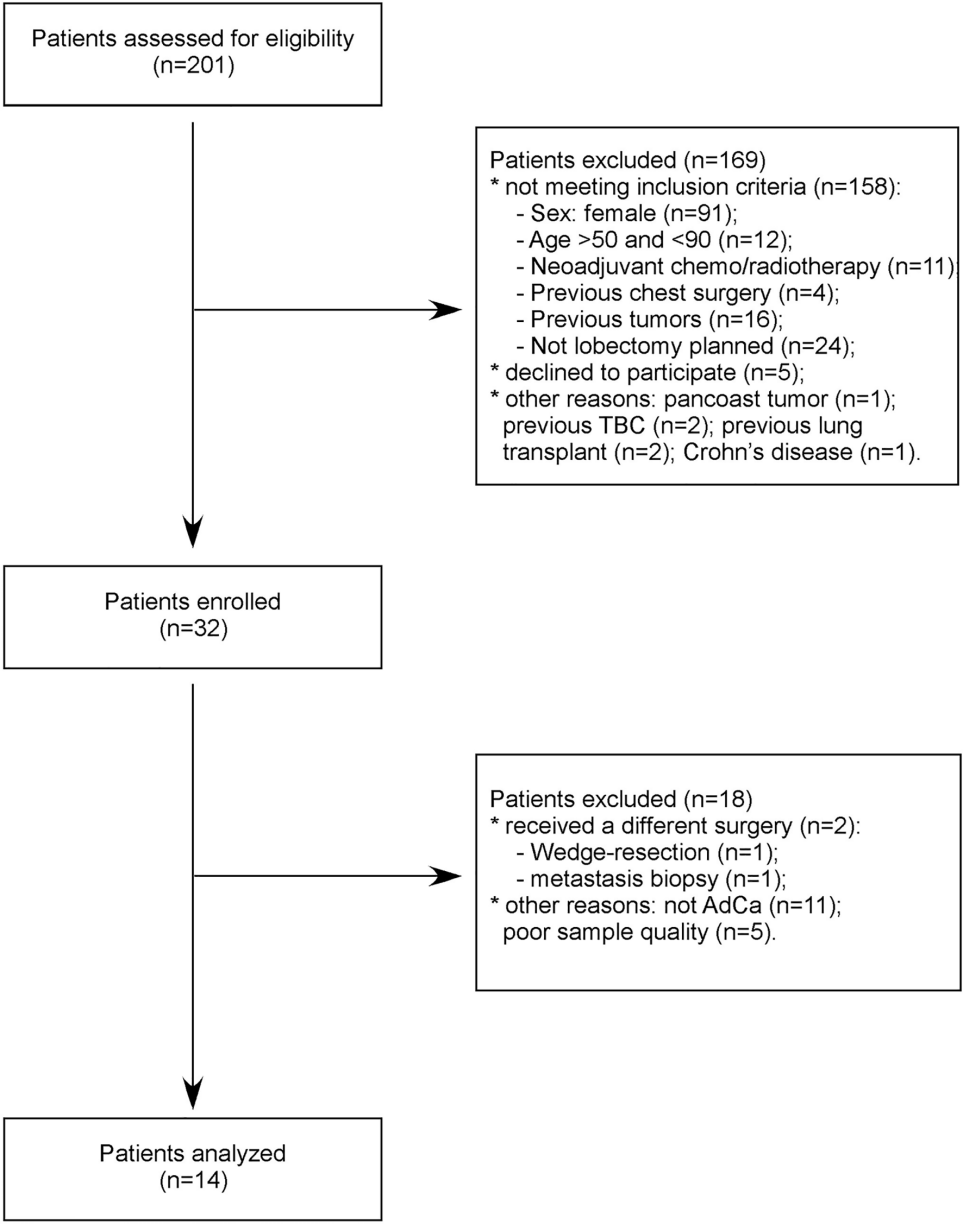
Patients’ selection flowchart.

From 2015 to 2016, we prospectively recruited a consecutive series of patients with early stage, i.e. stages I–II, AdCa and scheduled for pulmonary lobectomy at the Fondazione IRCCS Ca’ Granda—Ospedale Maggiore Policlinico. Inclusion criteria were sex male, age ≥50 years, current or former smokers, ability to tolerate general anesthesia and cardiopulmonary reserve to tolerate a lobectomy. Exclusion criteria were previous thoracic surgery or previous personal history of any cancer. Fourteen patients were included in the study (**Figure 1**). After the usual clinical and disease-specific preoperative assessment, patients underwent pulmonary lobectomy and systematic lymphadenectomy. Preoperative clinical data and postoperative histological report were recorded, anonymously, in a dedicated database (**Table 1**). One week before surgery, we collected EBC and plasma samples from all patients. As cancer-free controls, we collected EBC and plasma samples from nine age- and gender-matched voluntary subjects (**Supplementary Table 1**).

**Table 1:** Clinicopathological features of the 14 patients affected by AdCa.

Sample	Sex	Age	Histotype	TNM^1^	Smoke^2^
AdCa1	M	77	Adenocarcinoma	T1bN0	Previous
AdCa2	M	63	Adenocarcinoma	T1aN2	Yes
AdCa4	M	79	Adenocarcinoma	T2aN2	Previous
AdCa6	M	59	Adenocarcinoma	T2aN1	Previous
AdCa7	M	79	Adenocarcinoma	T2aN1	Previous
AdCa8	M	66	Adenocarcinoma	T2aN2	Previous
AdCa9	M	67	Adenocarcinoma	T1bN0	Yes
AdCa11	M	68	Adenocarcinoma	T2aN0	Previous
AdCa12	M	73	Adenocarcinoma	T1bN2	Previous
AdCa14	M	57	Adenocarcinoma	T2bN0	Previous
AdCa15	M	71	Adenocarcinoma	T2N2	Previous
AdCa16	M	65	Adenocarcinoma	T1aN0	Yes
AdCa18	M	80	Adenocarcinoma	T1bN0	Previous
AdCa19	M	71	Adenocarcinoma	T1aN0	Previous

1 All patients were M0 at surgery (*i.e.* no distant metastasis were present at the diagnosis).

2 The term previous, refers to at least 6 months before surgery.

The protocol was approved by the Ethics local Committee of Fondazione IRCCS Ca’ Granda-Ospedale Maggiore Policlinico, Milan, Italy (approval number: 879bis/11.12.2014) and was performed in agreement with the Helsinki Declaration. Informed consent was signed by each participant.

EBC- and plasmatic-miRNA profiles were also available for the previously described malignant pleural mesothelioma series (MPM; n = 23) and corresponding control subjects (asbestos-exposed, cancer-free individuals; n = 19) (12). For data validation we used a publicly available dataset of early stage NSCLCs and controls (GSE64591) annotated for gender, age and smoking habit (17). To remain strict to our study design, only males were included in the analysis. Accordingly, 86 NSCLCs and 71 controls were available.

### EBC and Blood Collection

Samples from each study participant included: I) EBC, collected and processed according to American Thoracic Society/European Respiratory Society recommendations (18), using the DECCS (Disposable Exhaled Condensate Collection System) through a transportable unit for use in research (Medivac—Parma, Italy) as previously described (19, 20); II) About 7.5 ml blood sample, collected in EDTA Vacutainer tubes (Becton Dickinson, Franklin Lakes, NJ, USA) that was centrifuged within 3 h from collection at 1,200*g* for 15 min to separate the plasma fraction from the blood cells.

### Tissue Microdissection and RNA Isolation

Tumor epithelium (TE), tumor-associated stroma (TS), non-neoplastic bronchial epithelium (BE) and alveolar parenchyma (A) were obtained by laser-assisted microdissection (Leica Microsystems, Milan, Italy) from formalin-fixed and paraffin embedded (FFPE) cancer and normal lung tissues of the enrolled patients (**Table 1**). Microdissection was performed as already described (21). Then, total RNA was isolated using the MasterPure RNA Purification Kit (Epicentre Biotechnologies, Madison, WI, USA) according to the supplier’s protocol. To assess quality and quantity, all samples were analyzed by a 2100 Bioanalyzer (Agilent Technologies, Santa Clara, CA) using the Agilent RNA 6000 Pico Kit.

### Extracellular Vesicles (EV) and miRNA Isolation

After collection, EBC and plasma samples were centrifuged three times at increasing speeds (1,000*g*, 2,000*g*, 3,000*g*) for 15 min at 4°C to remove cell debris and aggregates. Then, supernatants were ultracentrifuged using the BeckmanCoulter Optima-MAX-XP centrifuge (Beckman Coulter Life Sciences, Indianapolis, IN, USA) at 110,000×*g* for 75 min at 4°C and decanted. The EV pellet was kept at −80°C until use. miRNAs were isolated with the miRNeasy purification kit and the Rneasy MiniEluite spin column (all from Qiagen Hilden, Germany) to enrich for the miRNAs fraction, as suggested by the manufacturer. Finally, miRNAs were eluted in a final elution volume of 20 μl and stored at −80°C until processed for expression analysis.

### EV-miRNAs Profiling

Reverse transcription (RT) was performed using Megaplex RT Primers, Pool A v2.1 and Pool B v3.0, with the TaqMan Micro RNA Reverse Transcriptase Kit (Thermo Fisher Scientific, Waltham, MA, USA) in a C1000 Thermal Cycler (Biorad, Hercules, CA, USA) as previously described (22). Then, converted miRNAs were pre-amplified before being analyzed using the TaqMan OpenArray Human miRNA Panel, with QuantStudio AccuFill System Robot and the QuantStudio 12K Flex Real-Time PCR System (Thermo Fisher Scientific). This platform contains 754 unique miRNAs and four internal controls (ath-miR159a, RNU48, RNU44 and U6). miRNAs with a threshold cycle (Ct) value >28, an AmpScore <1.24, or undetectable were considered not amplified and their Ct value was set to 29. Further, miRNAs that were not detected in all plasma or EBC samples (n = 438 and 590, respectively) were excluded from the analysis. Accordingly, 316 and 164 miRNAs were available for plasma or EBC study. The NormFinder (23) algorithm was applied to choose the best normalization strategy. The global mean, was selected as the best normalization method for data normalization and miRNA quantification in EBC and plasma samples and the miRNA expression was quantified using the relative quantification 2^−ΔΔCt^ formula (24).

### Confirmation of miRNAs Expression by Individual qPCR

The top 11 miRNAs identified as associated with AdCa were validated in plasma and in tissue samples by qPCR performed in triplicate using a custom primers pool. The U6 snRNA assay was added for data normalization. All reagents and instrument were from Thermo Fisher Scientifics. For this analysis, miRNAs with a Ct value >38, an AmpScore <1.1 were considered undetectable and their Ct value was set to 40. miRNA expression was quantified using the relative quantification 2^−ΔΔCt^ (24).

### Statistical Analysis

Descriptive statistics were performed on all variables. Data are expressed as means ± SD or frequencies, as appropriate. To compare characteristics of the study patients by cases and controls we performed the chi-square test for categorical variables or the independent two-sample t-test for continuous variables. The mean value for each miRNA was calculated for AdCa and control groups and their ratio was used to obtain the Fold Change (FC). Then, miRNA expression values were log2 transformed to satisfy the linearity assumption. For each miRNA a logistic regression model adjusted for age, BMI (Body Mass Index) and smoking habits was run to assess miRNA discrimination between case and controls. The Odds Ratios (OR) were corrected for age, smoke and BMI and the standard error (SE) was computed as well. Due to the high number of comparisons, we applied a multiple comparison correction method based on the Benjamini–Hochberg False Discovery Rate (FDR) to calculate the FDR-adjusted p-value (p-FDR). In the screening analysis, a miRNA was considered to be differentially expressed if the p-value was <0.05, p-FDR was <0.25 and FC was <0.5 or >2. In the validation phase, miRNAs with raw p-value <0.05 were considered differentially expressed. To investigate the relationship between miRNA expression levels and type of tissues (A, BE, TE, TS) we performed linear mixed models for paired data. Analyses were performed using SAS 9.4 (SAS Institute, Cary, NC). To analyze EBC and plasma miRNAs levels in another type of cancer of the respiratory district, i.e. pleural mesothelioma, we used an already published dataset (12). Clustering analyses were performed either using the using the ComplexHeatmap package available within Bioconductor (https://bioconductor.org/packages/release/bioc/html/ComplexHeatmap.html) or the Heatmap tool available within GraphPad Prism software (Prism, La Jolla, CA, USA), as specified in each figure legend.

miRNA targets analysis was conducted using the multiMiR R package release 3.12 available within Bioconductor (http://multimir.ucdenver.edu), considering targets predicted by at least two algorithms. Then, predicted targets were imported in STRING and Gene Ontology analysis was performed to identify enriched Biological processes. The genome was used as background.

## Results

### miRNAs Analysis in EBC and Plasma Samples Revealed a Distinctive miRNAs Expression Profile in AdCa Patients Compared to Healthy Subjects

We started the analyses profiling 754 miRNAs in the EBC derived from the lung cohort. To limit the biological variability of circulating miRNA signatures, lung cancer patients included in this study were selected following stringent criteria (**Figure 1**). Indeed patients had to be males, between the ages of 55 and 90, current or former smokers and affected by a non-metastatic lung adenocarcinoma. Following these criteria, we enrolled a homogeneous group of 14 AdCa patients. Similarly, control subjects (n = 9) were sex-, age- and habits-matched with cases. For two AdCa subjects, EBC-miRNAs could not be analyzed because of poor RNA quality.

**Figure 2:**
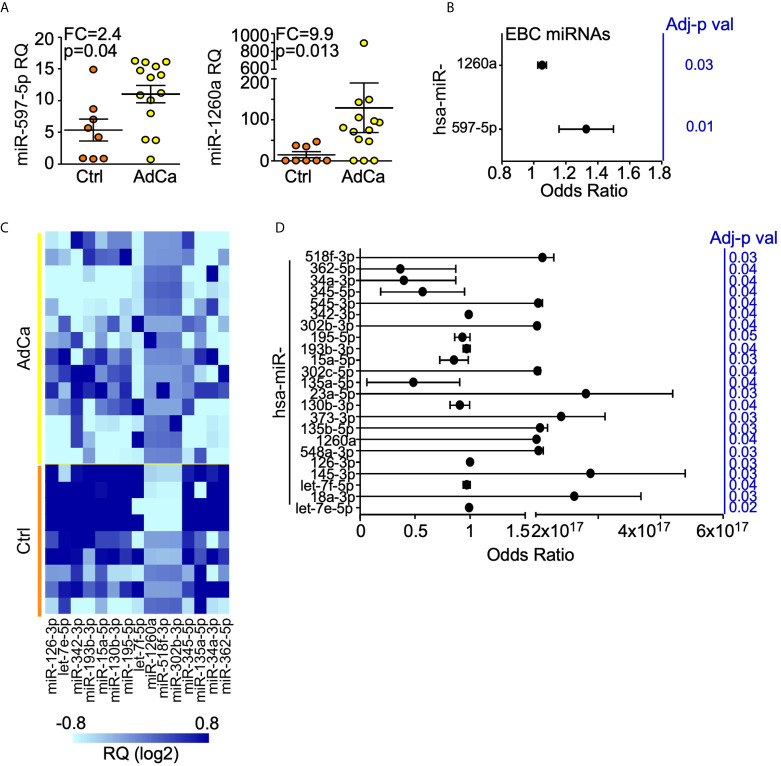
miRNAs expression profile in EBC and plasma from AdCa patients and healthy controls. **(A)** miR-597-5p and miR-1260a expression levels in the EBC of patients with AdCa or healthy controls (Ctrl). Each dot is a case; lines, mean with interquartile range; FDR-adjusted *p* val <0.25; FC, fold-change; RQ, relative quantity. **(B)** Diagnostic value of EBC-miRNAs in identifying subjects with lung AdCa. Forest plot showing the odds ratio for the EBC miRNA miR-597-5p and miR-1260a. Bars, 95% confidence interval. Odds Ratio was adjusted for age, smoke and BMI; *p* values are from logistic regression analysis. **(C)** Heatmap of the plasma miRNAs differentially expressed (FC >2; FDR-adjusted p val <0.25) between AdCa patients and control (Ctrl) subjects. The heatmap was generated with GraphPad Prism software. **(D)** Diagnostic value of plasma-miRNAs in identifying subjects with lung AdCa. Forest plot showing the odds ratio for the indicated plasma miRNAs. Bars, 95% confidence interval. Odds Ratio was adjusted for age, smoke and BMI; *p* values are from logistic regression analysis; FC, fold change.

One hundred and sixty-four (22%) miRNAs resulted expressed in at least one sample. When we performed an unsupervised hierarchical clustering, miRNAs expression levels showed a distinctive signature in patients compared to controls (**Supplementary Figure 1A**). In particular, miR-597-5p and miR-1260a were over-expressed in patients compared to healthy subjects (FC = 2.4 and 9.9, respectively; **Figure 2A**) and their levels were significantly associated with lung cancer (OR = 1.31, SE = 0.11, p = 0.019 and OR = 1.1, SE = 0.02, p = 0.031, for miR-597-5p and miR-1260a respectively; **Figure 2B**).

The same analysis was performed for the plasma samples. This time, 316 miRNAs (42%) were expressed in at least one sample (**Supplementary Figure 1B**), of which twelve miRNAs (miR-15a-5p, -34a-3p, -126-3p, -130b-3p, -135a-5p, -193b-3p, -195-5p, -342-3p, -345-5p, -362-5p, let-7e-5p and let-7f-5p) showed at least 2-fold down-regulation, whereas miR-302b-3p, -518f-3p and -1260a were at least 4-folds up-regulated in patients compared to healthy subjects (**Figure 2C**). Lastly, 23 plasma-miRNAs were significantly associated with lung cancer presence (**Figure 2D**).

At validation, we could confirm a significant deregulation of plasmatic miR-130b-3p, miR-302b-3p and miR-518f-3p in patients compared to controls (**Table 2**). A trend for let-7f-5p, miR-345-5p, and miR-362-5p under-expression in AdCa compared with controls could be detected along with up-regulation of miR-1260a (**Table 2**). No miRNAs was associated with patients’ clinicopatological features, including tumor size or nodal metastasis.

**Table 2:** Validation analysis: the expression level and odds ratio (OR) of the plasmatic miRNAs is reported^3^.

miRNA	CTRL (RQ mean)	AdCa (RQ mean)	OR	SE	P value
hsa-let-7f-5p	11.7	7.5	0.301	0.591	0.04
hsa-miR-130b-3p	6.7	2.3	0.410	0.430	0.03
hsa-miR-135a-5p	0.1	0.1	0.649	0.288	0.13
hsa-miR-302b-3p	1.07 × 10^−5^	3.40 × 10^−5^	2.849	0.595	0.08
hsa-miR-342-3p	292.2	359.7	0.561	0.519	0.26
hsa-miR-345-5p	4.4	3.0	0.406	0.488	0.06
hsa-miR-362-5p	0.5	0.3	0.390	0.487	0.05
hsa-miR-518f-3p	0.006	0.07	1.030	0.085	0.73
hsa-miR-597-5p	0.086	0.01	0.852	0.092	0.08
hsa-miR-1260a	8.2	13.8	1.615	0.644	0.46

^3^OR was adjusted for age, smoking and body mass index (BMI). P values refer to the OR estimation using a linear regression model.

Overall, from this miRNA screening we could detect that miR-1260a was significantly higher in patients compared with healthy controls both in EBC and plasma analyses, suggesting that it could be a circulating biomarker of lung AdCa.

### miRNAs Analysis in Tumor and Normal Lung Tissues Components

**Figure 3:**
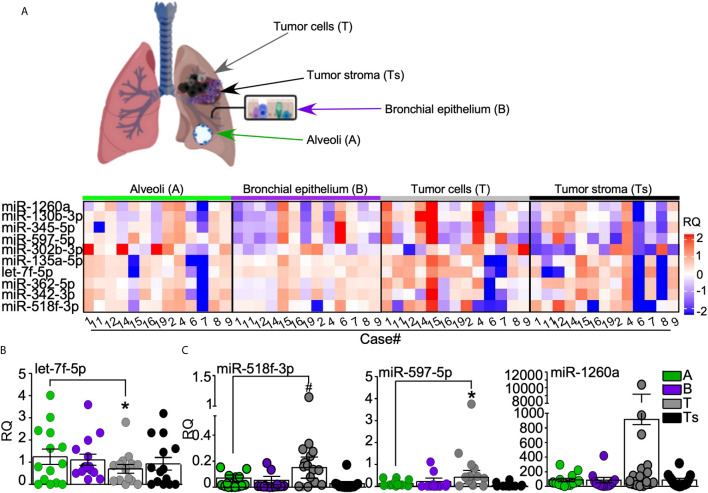
miRNAs validation analysis. **(A)** Normal bronchial epithelium (B), alveoli (A) as well as lung tumor cells (T) and tumor-surrounding stroma (Ts) were laser-assisted microdissected from surgical specimens of 13 out of the 14 AdCa patients. Heatmap (generated with ComplexHeatmap package) shows the expression levels of the ten selected miRNAs in each case. *Upper panel*, representative images of the analyzed tissues. **(B, C)** The expression of the differentially regulated miRNAs in tumor cells is shown, with miRNA let-7f-5p being down regulated **(B)**, while miR-518f-3p, miR-597-5p, and miR-1260a **(C)** being up-regulated in lung tumor cells. Each dot is a sample; bars, mean ± SD. *p = 0.04, ^#^p = 0.03 by Mann–Whitney U test.

In order to investigate the origin of the most relevant miRNAs deregulated in plasma and EBC of AdCa patients, we isolated by laser-microdissection different tumor cellular compartments, namely tumor epithelial cells (T) and tumor-associated stroma (Ts), as well as normal lung tissues, namely bronchial epithelium (B) and alveoli (A; **Supplementary Figure 2**) from archival blocks of our patients’ series. Then, we analyzed the expression levels of the ten miRNAs (miR-130b-3p, -135a-5p, -302b-3p, -342-3p, -345-5p, -362-5p, -518f-3p, -597-5p, -1260a and let-7f-5p) in the microdissected tissues. This analysis highlighted distinctive miRNA profiles in the different tissues (**Figure 3A**). In line with the miRNA screening, the tumor suppressor miRNA let-7f-5p showed higher expression in normal tissues than in tumors (**Figure 3B**), whereas miR-518f-3p, miR-597-5p and -1260a, all up-regulated in plasma and EBC of AdCa, were more abundantly expressed in tumor cells than in the surrounding stroma or normal tissues (**Figure 3C**).

This experiment suggests the cell of origin of the differentially expressed miRNAs, and supports the concept that miR-1260a, miR-518f-3p and miR-597-5p could act as onco-miR in lung cancer environments.

### Analysis of the Circulating miRNAs Signature in an Independent Series of Lung AdCa and in Patients Affected by Malignant Pleural Mesothelioma

To confirm if the identified plasmatic miRNA signature could be relevant and useful to identify male patients with lung adenocarcinoma, we used a publicly available dataset with annotated clinical information and miRNA profiles (17). From this analysis, we could identify that the plasmatic miRNAs, let-7f-5p and miR-518f-3p showed a trend in identifying patients with lung AdCa (**Supplementary Table 2**), as we found in the discovery phase.

**Figure 4:**
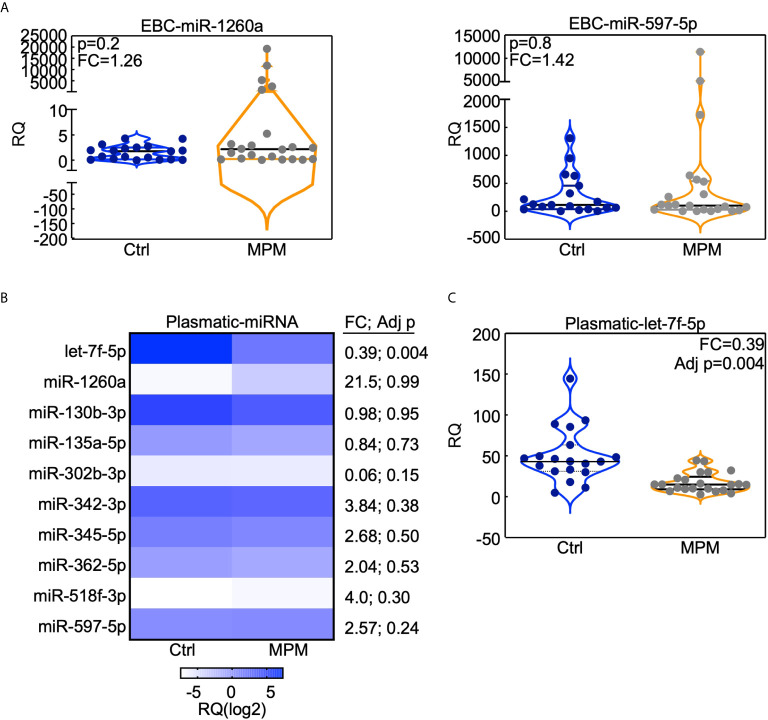
miRNAs analysis in the MPM cohort. The indicated miRNAs were analyzed in the EBC **(A)** or in the plasma **(B, C)** obtained from 23 MPMs and 19 cancer-free subjects who were previously exposed to asbestos (Ctrl). **(B)** Heatmap (generated with GraphPad Prism software) indicates the expression level of the indicated plasmatic miRNA in MPM or control subjects. **(A, C)** Data are presented with violin plots where each case is a dot and lines indicate median with interquartile range. RQ, Relative quantity; FC, fold change; Adj p, FDR-adjusted p value.

To investigate whether our miRNA signature was specific for lung adenocarcinomas, we investigated the levels of the ten miRNAs in the EBC and plasma of the malignant pleural mesothelioma cohort (12). Neither miR-1260a, nor miR518f-3p or miR-597-5p was modulated in the EBC of the MPM patients with respect to control individuals (**Figure 4A** and **Supplementary Figure 3**). On the contrary, when we analyzed plasmatic miRNAs (**Figure 4B**), let-7f-5p levels were significantly down-regulated in the circulation of MPM patients compared to controls (**Figure 4C**), similarly to what observed for the lung AdCa cohort.

Therefore, we could conclude that the 3-miRNAs signature composed by miR-1260a, miR-597-5p and miR-518f-3p, could be specific for lung AdCa disease, whereas let-7f-5p decrease characterizes also other neoplasia of the respiratory district such as malignant pleural mesothelioma.

### miRNAs Targets Analysis

In an attempt to preliminary speculate on potential signaling affected by the identified AdCa-upregulated miRNAs, namely miR-518f-3p, -597-5p, and miR-1260a, we performed an *in silico* analysis of predicted targets and potentially affected pathways. This analysis showed that tumor bioenergetics was the most affected signaling by the 3-miRNAs signature (**Supplementary Tables 3**, **4**). This analysis, although *in silico*, suggests that circulating onco-miRNAs might alter cell metabolism participating in promoting a pro-tumorigenic environment also in the lung.

## Discussion

Our study investigated the expression of 754 miRNAs in EBC and plasma samples of patients affected by AdCa and healthy control subjects. Both the EBC and plasma-miRNAs analyses highlighted a distinctive expression profile in patients compared to healthy subjects. In particular, miR-597-5p and -1260a were up-regulated in EBC of patients compared to controls and were associated with lung cancer presence. Interestingly, miR-1260a was upregulated also in the plasma of AdCa patients compared with controls.

The four miRNAs (let-7f-5p, miR-518f-3p, -597-5p and -1260a) signature resulted particularly interesting to discriminate patients affected by AdCa. Further, decreased le-7f-5p and increased miR-518f-3p levels were marginally associated to lung cancer in an independent series of male subjects (17). To test the specificity of this signature in identifying lung cancer patients, we analyzed circulating miRNAs in a second neoplasia of the respiratory district, namely the malignant pleural mesothelioma. This analysis showed that the onco-suppressor miRNA let-7f-5p was decreased also in the circulation of mesothelioma patients, highlighting the broad repression of this miRNA in different cancer settings. Indeed, members of the let-7 miRNAs family have been found down-regulated in different types of tumors targeting several oncogenes such as Ras, HMGA2 and cMyc (25).

**Figure 5:**
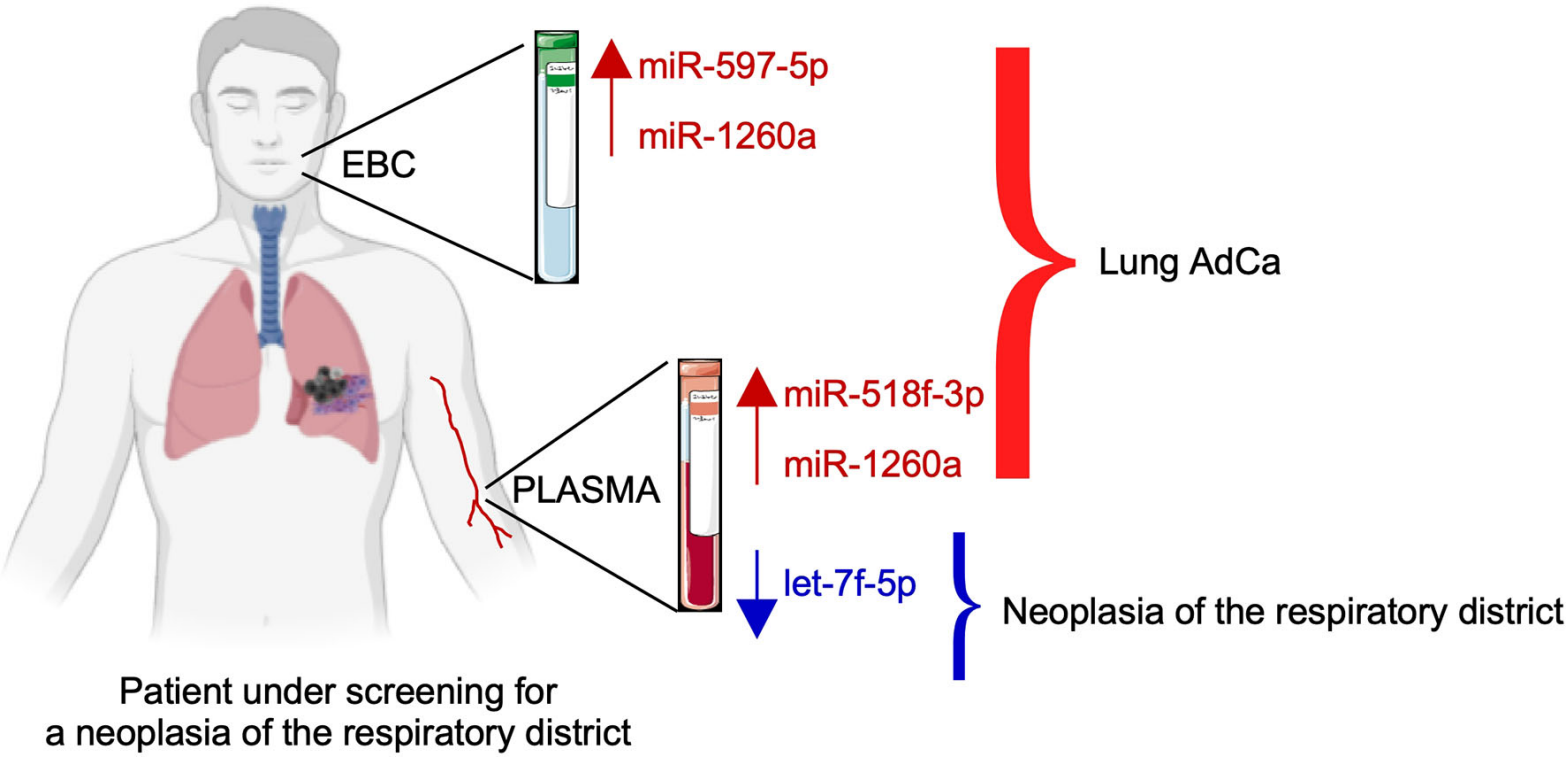
Schematic of the proposed model of the three-miRNAs signature as an adjunct tool to screen subjects for lung cancer presence.

On the other hand, miR-518f-3p, miR-597 and miR-1260 levels were unchanged in the plasma of mesothelioma patients compared with controls, and therefore, their up-regulation was specific for lung AdCa (**Figure 5**).

Further, we could document that these miRNAs are predominantly expressed by tumor epithelial cells and not by the stromal microenvironment. In line with its onco-suppressor role, let-7f-5p was more expressed in normal alveolar cells than in the tumor compartments.

Finally, *in silico* analysis showed that these miRNAs target several transcripts involved in tumor bioenergetics. Data from literature, document that miR-1260 is correlated with poor prognosis in breast cancer (26) and in neuroblastoma (27), whereas miR-518f belongs to the oncogenic C19MC miRNA cluster (28).

A limitation of our study is the small number of patients enrolled. In order to reduce biological variability and to improve the results accuracy, we applied stringent criteria for patients’ selection. On the other hand, we profiled two types of circulating miRNAs (EBC and plasma), and we verified the signatures in another cancer of the respiratory district, i.e. pleural mesothelioma to firstly provide a preliminary snapshot of non-invasive biomarkers of thoracic cancers that could implement differential diagnosis besides early detection.

The identification of biomarkers able to recognize the presence of lung cancer using non-invasive methods in addition to imaging analysis is one of the cancer research goals. Different studies have been focused on the detection of miRNAs in biological fluids, because the liquid biopsy is a valid, minimal-invasive method to explore pathological biomarkers (10, 11, 16, 29, 30). Several studies highlighted the potentiality of miRNAs detection in plasma, but few works investigated miRNAs in EBC (16, 30). EBC collection is a non-invasive and reproducible procedure which allows the analysis of molecules derived from lungs and lower respiratory tract (16, 30). Recently, a study reported that elevated miR-21 expression together with decreased miR-486 in EBC and plasma discriminates NSCLC patients from healthy subjects (16). This indicates that circulating miRNAs may have a diagnostic value and may serve as early non-invasive biomarkers in adjunct to the current screening methods. Nevertheless, to eventually confirm the use of miRNA as non-invasive biomarkers of lung AdCa or MPM in addition to LDCT in the clinical setting, larger cohort of patients are needed together with prospective studies on pre-diagnostic samples from longitudinally followed cohorts of at-risk subjects.

Altogether, our results and the recent literature support the use of the volatile biopsy as a reliable method to identify biomarkers useful in the clinical settings and propose a combined plasma/EBC 3-miRNAs signature to implement the screening protocols for cancers of the respiratory district.

## Data Availability Statement

The raw data supporting the conclusions of this article will be made available by the authors, without undue reservation.

## Ethics Statement

The studies involving human participants were reviewed and approved by the Ethics Committee of Fondazione IRCCS Ca’ Granda-Ospedale Maggiore Policlinico, Milan, Italy (approval number: 879bis/11.12.2014). The patients/participants provided their written informed consent to participate in this study.

## Author Contributions

MM, VM, MN, and ACP—patient selection. AF, CF, VB, VV, and AP—conceptualization and methodology. AF, CF, LD, AT, VM, NF, and MM—investigation. VM, MM, ACP, VB, and MN—data curation. AT, MM, ACP, CF, and NF—data analysis and interpretation. AF, NF, VV, and AP—manuscript drafting. VV and AP—manuscript finalization. All authors contributed to the article and approved the submitted version.

## Funding

This work was supported by the Italian Minister of Health to VV (GR2011-02351626) and by Fondazione IRCCS Ca’ Granda Project Competition program 2015/16 to AP.

## Conflict of Interest

The authors declare that the research was conducted in the absence of any commercial or financial relationships that could be construed as a potential conflict of interest.

## Footnotes

1 All patients were M0 at surgery (*i.e.* no distant metastasis were present at the diagnosis).

2 The term previous, refers to at least 6 months before surgery.

## Abbreviations

A, alveoli; AdCa, Lung Adenocarcinonma; BMI, Body Mass Index; B, Bronchial epithelium; DECCS, Disposable Exhaled Condensate Collection System; EBC, Exhaled Breath Condensate; EV, Extracellular Vesicles; FC, Fold Change; FDR, False Discovery Rate; FFPE, Formalin-Fixed and Paraffin Embedded; miRNA, microRNA; NSCLC, Non-Small Cell Lung Cancer; RT, Reverse Transcription; TBC, Tubercolosis; T, Tumor Epithelium; Ts, Tumor Stroma.
