# Supplementary material for: An EBC/Plasma miRNA Signature Discriminates Lung Adenocarcinomas From Pleural Mesothelioma and Healthy Controls
Source: Front Oncol. 2021 Jun 15;11:643280. doi: 10.3389/fonc.2021.643280 (PMC8239300; doi:10.3389/fonc.2021.643280)
Supplement: Supplementary file 1 [file DataSheet_1.docx]

**An EBC/plasma miRNA signature discriminates lung adenocarcinomas from pleural mesothelioma and healthy controls**

Alice Faversani, Chiara Favero, Laura Dioni, Angela Cecilia Pesatori, Valentina Bollati, Matteo Montoli, Valeria Musso, Andrea Terrasi, Nicola Fusco, Mario Nosotti, Valentina Vaira and Alessandro Palleschi

**Supplementary Material**

Supplementary Material includes 2 Supplementary Figures and 2 Supplementary Tables.

**Supplementary Figures**

**Supplementary Fig. 1.** miRNA expression profile in EBC and plasma derived from patients affected by AdCa and healthy controls. A) Unsupervised hierarchical clustering of EBC miRNAs (A) or plasma-derived miRNAs (B) from AdCa patients and healthy controls. Heatmaps were generated in the R environment using the ComplexHeatmap tool.

**
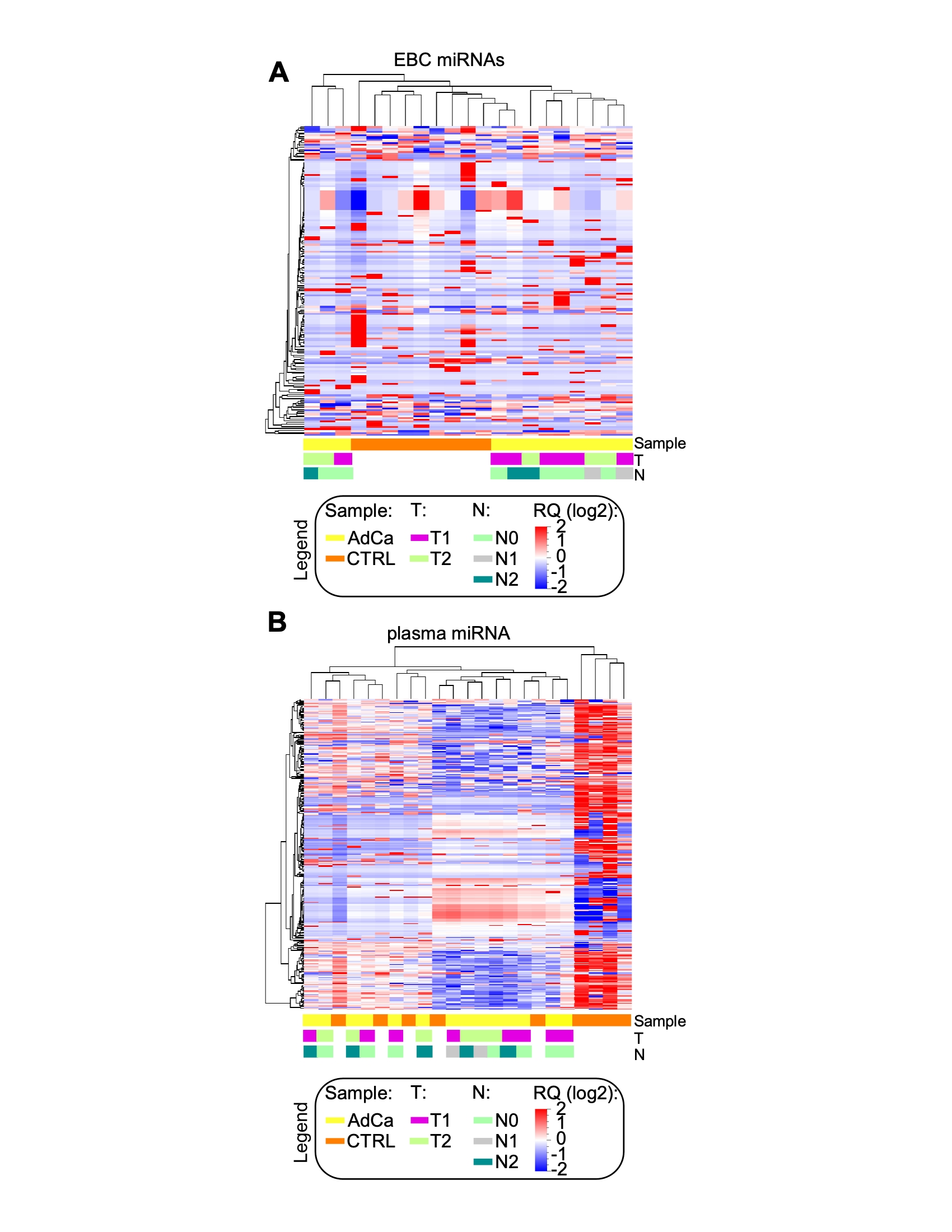
**

**Supplementary Fig. 2.** miRNAs analysis in microdissected lung tissues. Normal bronchial epithelium, alveoli and two tumor lung components, i.e. tumor epithelial cells and stroma, were isolated by laser-assisted microdissection from 13 patients affected by lung AdCa. miRNAs were then purified from these tissues and their expression was quantified by qPCR. Representative images of laser-assisted microdissected tissues are shown.

**
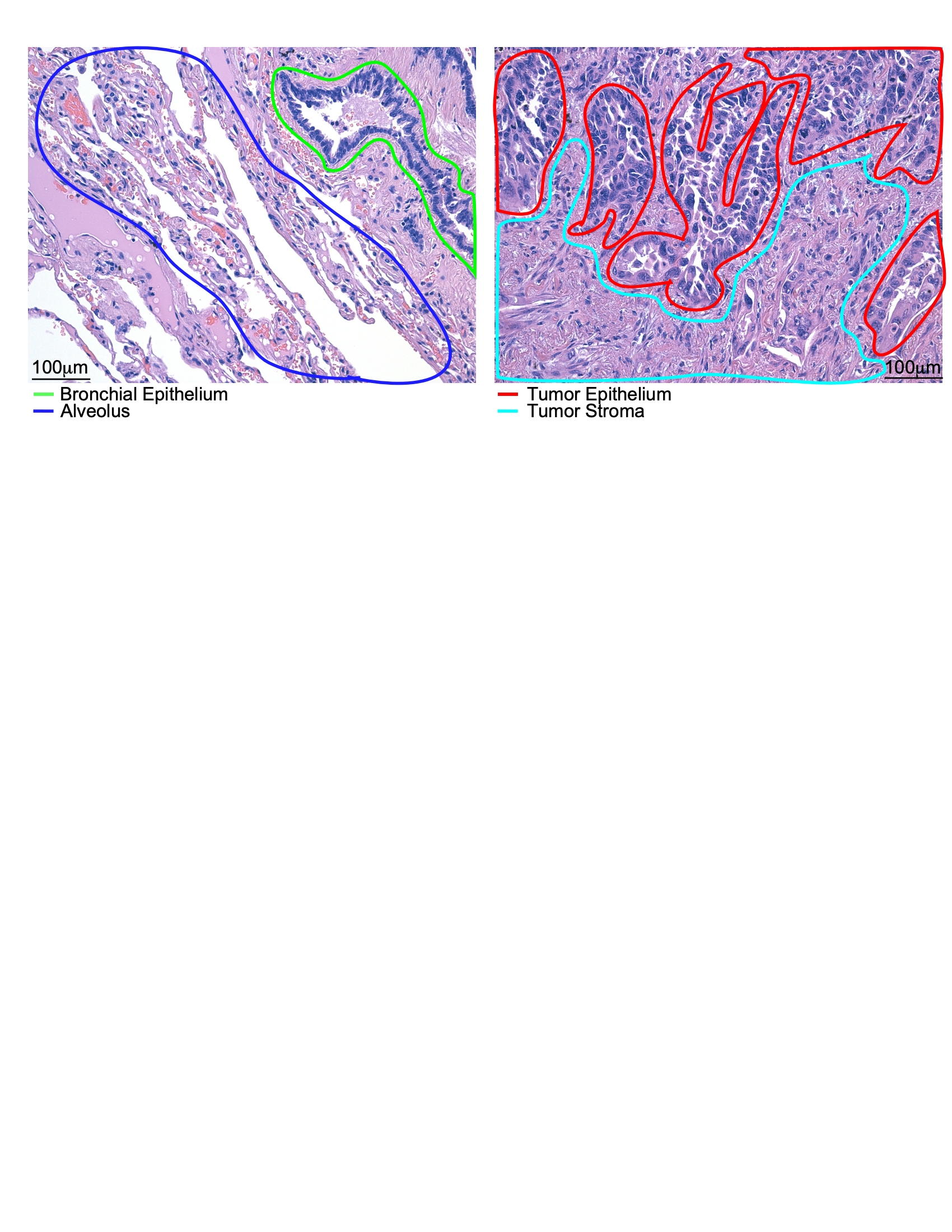
**

**Supplementary Fig. 3. miR-518f-3p evaluation in the EBC of MPM patients and controls.** The indicated miRNA was evaluated in the EBC of 23 MPMs and 19 cancer-free subjects who were previously exposed to asbestos (Ctrl). Data are presented with box plot where each case is a dot and lines indicate median with interquartile range. RQ, Relative quantity; FC, fold change.

**
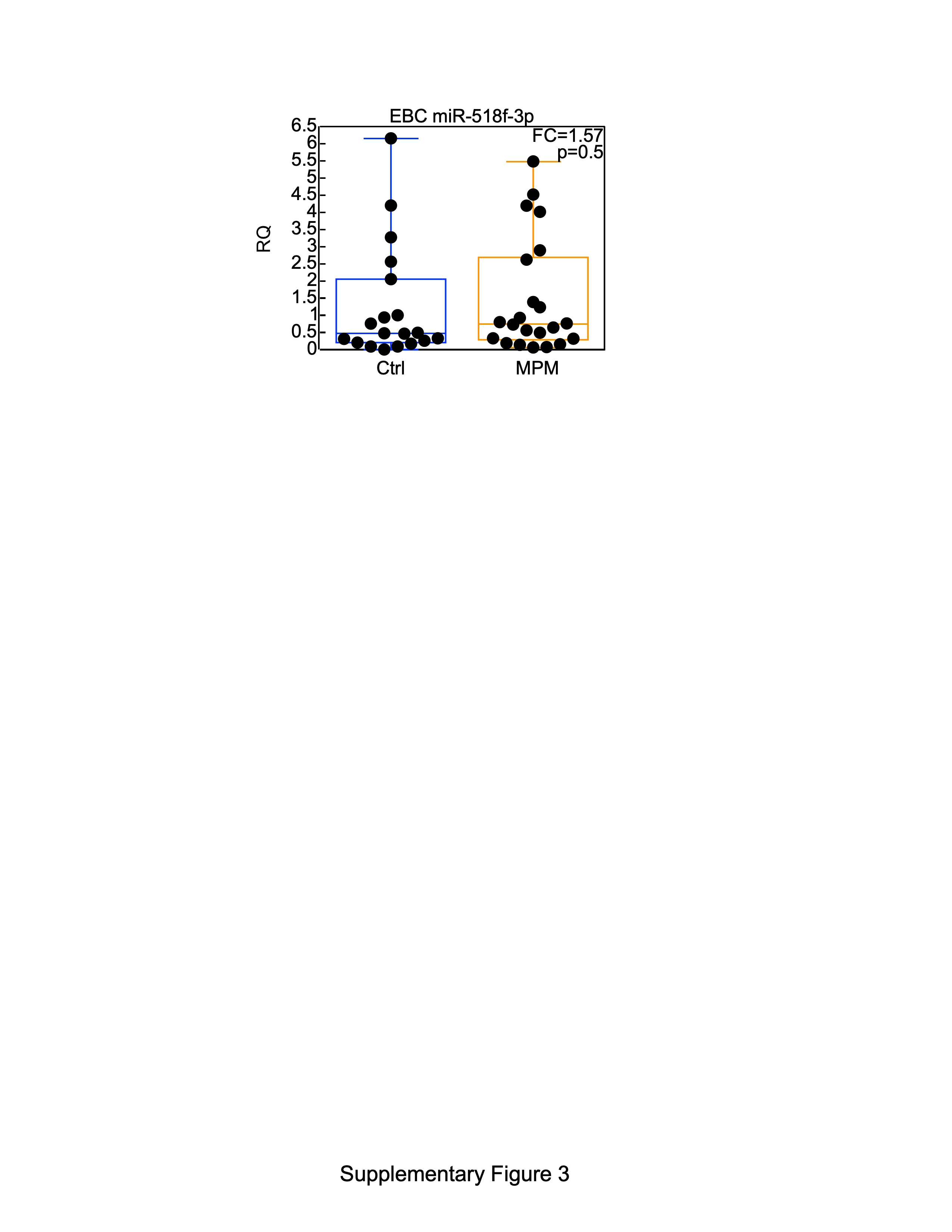
**

**Supplementary Tables**

**Supplementary Table 1**: Description of cases and controls features. All patients are males.

|  | Cases (n=14) | Controls (n=9) | p-Value |
| --- | --- | --- | --- |
| Age (Average ± SD) | 69 ± 7 | 67 ± 7 | 0.775 |
| BMI (Average ± SD) | 24.7 ± 2.7 | 26.7 ± 3.1 | 0.841 |
| Smoke |  |  | 0.611 |
| No | 0 | 0 |  |
| Previous | 10 (71%) | 6 (66.7%) |  |
| Yes | 2 (14.3%) | 3 (33.3%) |  |
| *NA* | 2 (14.3%) | 0 |  |

**Supplementary Table 2.** Validation of the plasmatic miRNA signature in a second independent cohort (Geo accession number GSE64591) of NSCLC (n=86) and controls (n=71). Only male subjects were considered, and the analysis was adjusted for age and smoking habit.

| **miRNA^[[1]](#footnote-1)^** | **FC NSCLC/controls** | **OR** | **95% CI** | | ***P*-value** | **adj *P*-value** |
| --- | --- | --- | --- | --- | --- | --- |
| let-7e-5p | 0.96 | 1.00033 | 1.0000 | 1.00 | 0.069 | 0.989 |
| hsa-let-7f-5p | 0.92 | 1.00000 | 1.0000 | 1.00 | 0.043 | 0.399 |
| mir-126-3p | 0.95 | 1.00031 | 1.0001 | 1.00 | 0.004 | 0.118 |
| miR-1260a | 1.02 | 1.00000 | 1.0000 | 1.00 | 0.781 | 0.999 |
| miR-130b-3p | 0.95 | 1.00000 | 1.0000 | 1.00 | 0.862 | 0.999 |
| miR-130b-5p | 0.97 | 1.00270 | 0.9975 | 1.01 | 0.313 | 0.999 |
| miR-135a-5p | not expressed |  |  |  |  |  |
| miR-135b-5p | not expressed |  |  |  |  |  |
| mir-145-5p | 0.96 | 1.00083 | 0.9934 | 1.01 | 0.827 | 0.999 |
| miR-15a-5p | 0.95 | 1.00000 | 1.0000 | 1.00 | 0.533 | 0.999 |
| miR-18a-3p | 1.09 | 1.00411 | 0.9997 | 1.01 | 0.070 | 0.507 |
| miR-193b-3p | 0.98 | 1.18900 | 0.7371 | 1.92 | 0.478 | 0.999 |
| miR-195-5p | 0.98 | 1.00085 | 0.9994 | 1.00 | 0.236 | 0.980 |
| miR-23a-5p | 0.97 | 0.99998 | 0.9995 | 1.00 | 0.934 | 0.999 |
| miR-302b-3p | not expressed |  |  |  |  |  |
| miR-302c-5p | 0.99 | 0.99999 | 0.9998 | 1.00 | 0.941 | 0.999 |
| miR-342-3p | 0.97 | 1.00012 | 0.9999 | 1.00 | 0.170 | 0.847 |
| miR-345-5p | 0.97 | 1.00505 | 0.9973 | 1.01 | 0.203 | 0.936 |
| mir-34a-3p | 1.00 | 1.00000 | 1.0000 | 1.00 | 0.575 | 0.999 |
| miR-362-5p | 0.98 | 1.00000 | 0.9999 | 1.00 | 0.984 | 0.999 |
| miR-373-3p | not expressed |  |  |  |  |  |
| miR-518f-3p | 1.1 | 1.00000 | 1.0000 | 1.00 | 0.015 | 0.200 |
| miR-545-3p | 1.03 | 1.00000 | 1.0000 | 1.00 | 0.602 | 0.999 |
| miR-548a-3p | 1.00 | 0.95185 | 0.8774 | 1.03 | 0.235 | 0.980 |

**Supplementary Table 3**. Prediction analysis of the targets and associated pathways of the three upregulated miRNAs miR-1260a, miR-518f-3p and and miR-597-5p was performed with the multiMiR R package (the script is provided as a Supplementary Data 1). The number of times a gene was identified as predicted target by the indicated algorithm is shown, together with the total number of algorithm that predicted the specific interaction (predicted.sum column).

| mature_mirna_acc | mature_mirna_id | target_symbol | target_entrez | diana_microt | microcosm | miranda | mirdb | pictar | pita | predicted.sum |
| --- | --- | --- | --- | --- | --- | --- | --- | --- | --- | --- |
| MIMAT0005911 | hsa-miR-1260a | AGO1 | 26523 | 1 | 0 | 0 | 3 | 1 | 2 | 4 |
| MIMAT0005911 | hsa-miR-1260a | CAP1 | 10487 | 1 | 0 | 1 | 1 | 0 | 1 | 4 |
| MIMAT0005911 | hsa-miR-1260a | LARP1B | 55132 | 0 | 0 | 2 | 2 | 1 | 1 | 4 |
| MIMAT0005911 | hsa-miR-1260a | PABPN1 | 8106 | 1 | 0 | 1 | 0 | 1 | 2 | 4 |
| MIMAT0005911 | hsa-miR-1260a | PTBP2 | 58155 | 1 | 0 | 0 | 2 | 1 | 1 | 4 |
| MIMAT0005911 | hsa-miR-1260a | TARDBP | 23435 | 1 | 0 | 0 | 1 | 1 | 1 | 4 |
| MIMAT0005911 | hsa-miR-1260a | ZNF302 | 55900 | 1 | 0 | 0 | 1 | 1 | 1 | 4 |
| MIMAT0002842 | hsa-miR-518f-3p | PTPRU | 10076 | 1 | 0 | 1 | 0 | 0 | 1 | 3 |
| MIMAT0002842 | hsa-miR-518f-3p | ZNF608 | 57507 | 0 | 1 | 2 | 0 | 0 | 1 | 3 |
| MIMAT0003265 | hsa-miR-597-5p | AMELX | 265 | 0 | 1 | 1 | 0 | 0 | 1 | 3 |
| MIMAT0003265 | hsa-miR-597-5p | CACNB2 | 783 | 1 | 0 | 1 | 0 | 0 | 1 | 3 |
| MIMAT0003265 | hsa-miR-597-5p | CARTPT | 9607 | 1 | 0 | 1 | 1 | 0 | 0 | 3 |
| MIMAT0003265 | hsa-miR-597-5p | DDX6 | 1656 | 1 | 0 | 0 | 1 | 0 | 1 | 3 |
| MIMAT0003265 | hsa-miR-597-5p | EIF4E3 | 317649 | 1 | 0 | 0 | 1 | 0 | 1 | 3 |
| MIMAT0003265 | hsa-miR-597-5p | FUT8 | 2530 | 1 | 1 | 1 | 0 | 0 | 0 | 3 |
| MIMAT0003265 | hsa-miR-597-5p | G3BP2 | 9908 | 1 | 0 | 1 | 1 | 0 | 0 | 3 |
| MIMAT0003265 | hsa-miR-597-5p | HSPA5 | 3309 | 1 | 0 | 1 | 0 | 0 | 1 | 3 |
| MIMAT0003265 | hsa-miR-597-5p | IL6ST | 3572 | 1 | 0 | 1 | 0 | 0 | 1 | 3 |
| MIMAT0003265 | hsa-miR-597-5p | JAK1 | 3716 | 1 | 1 | 0 | 1 | 0 | 0 | 3 |
| MIMAT0003265 | hsa-miR-597-5p | LARP4 | 113251 | 1 | 0 | 0 | 1 | 1 | 0 | 3 |
| MIMAT0003265 | hsa-miR-597-5p | MOAP1 | 64112 | 1 | 0 | 1 | 1 | 0 | 0 | 3 |
| MIMAT0003265 | hsa-miR-597-5p | NRXN3 | 9369 | 1 | 0 | 1 | 0 | 0 | 1 | 3 |
| MIMAT0003265 | hsa-miR-597-5p | ORAI1 | 84876 | 1 | 1 | 1 | 0 | 0 | 0 | 3 |
| MIMAT0003265 | hsa-miR-597-5p | TEAD1 | 7003 | 1 | 0 | 0 | 1 | 0 | 1 | 3 |
| MIMAT0003265 | hsa-miR-597-5p | XPO7 | 23039 | 1 | 0 | 2 | 0 | 0 | 1 | 3 |
| MIMAT0005911 | hsa-miR-1260a | ATF6B | 1388 | 1 | 0 | 0 | 1 | 1 | 0 | 3 |
| MIMAT0005911 | hsa-miR-1260a | BTBD11 | 121551 | 0 | 0 | 1 | 0 | 1 | 1 | 3 |
| MIMAT0005911 | hsa-miR-1260a | DLG2 | 1740 | 1 | 0 | 0 | 0 | 1 | 1 | 3 |
| MIMAT0005911 | hsa-miR-1260a | DNMT3A | 1788 | 1 | 0 | 0 | 0 | 1 | 2 | 3 |
| MIMAT0005911 | hsa-miR-1260a | DYRK1A | 1859 | 1 | 0 | 0 | 0 | 3 | 1 | 3 |
| MIMAT0005911 | hsa-miR-1260a | FAM120C | 54954 | 1 | 0 | 0 | 1 | 1 | 0 | 3 |
| MIMAT0005911 | hsa-miR-1260a | GDI1 | 2664 | 1 | 0 | 0 | 1 | 1 | 0 | 3 |
| MIMAT0005911 | hsa-miR-1260a | GLP1R | 2740 | 1 | 0 | 0 | 1 | 1 | 0 | 3 |
| MIMAT0005911 | hsa-miR-1260a | HECTD1 | 25831 | 1 | 0 | 1 | 0 | 1 | 0 | 3 |
| MIMAT0005911 | hsa-miR-1260a | IGF2BP1 | 10642 | 0 | 0 | 0 | 1 | 1 | 1 | 3 |
| MIMAT0005911 | hsa-miR-1260a | RELT | 84957 | 1 | 0 | 1 | 0 | 1 | 0 | 3 |
| MIMAT0005911 | hsa-miR-1260a | U2SURP | 23350 | 0 | 0 | 0 | 1 | 1 | 1 | 3 |
| MIMAT0005911 | hsa-miR-1260a | UBTD2 | 92181 | 0 | 0 | 1 | 1 | 0 | 1 | 3 |
| MIMAT0005911 | hsa-miR-1260a | XKR6 | 286046 | 0 | 0 | 1 | 1 | 1 | 0 | 3 |
| MIMAT0005911 | hsa-miR-1260a | ZNF268 | 10795 | 1 | 0 | 0 | 3 | 2 | 0 | 3 |
| MIMAT0005911 | hsa-miR-1260a | ZNF559 | 84527 | 1 | 0 | 0 | 1 | 2 | 0 | 3 |
| MIMAT0005911 | hsa-miR-1260a | ZNF594 | 84622 | 1 | 0 | 0 | 1 | 0 | 1 | 3 |
| MIMAT0002842 | hsa-miR-518f-3p | MARCH7 | 51617 | 1 | 0 | 1 | 0 | 0 | 0 | 2 |
| MIMAT0002842 | hsa-miR-518f-3p | AKR1B1 | 231 | 0 | 1 | 0 | 0 | 0 | 1 | 2 |
| MIMAT0002842 | hsa-miR-518f-3p | C1orf43 | 25912 | 0 | 1 | 1 | 0 | 0 | 0 | 2 |
| MIMAT0002842 | hsa-miR-518f-3p | CPEB1 | 64506 | 0 | 0 | 1 | 0 | 0 | 1 | 2 |
| MIMAT0002842 | hsa-miR-518f-3p | CYFIP1 | 23191 | 0 | 1 | 0 | 0 | 0 | 1 | 2 |
| MIMAT0002842 | hsa-miR-518f-3p | FBXO3 | 26273 | 0 | 0 | 1 | 1 | 0 | 0 | 2 |
| MIMAT0002842 | hsa-miR-518f-3p | HERPUD1 | 9709 | 0 | 1 | 0 | 0 | 0 | 1 | 2 |
| MIMAT0002842 | hsa-miR-518f-3p | KCNK12 | 56660 | 0 | 1 | 0 | 0 | 0 | 1 | 2 |
| MIMAT0002842 | hsa-miR-518f-3p | LSM5 | 23658 | 0 | 1 | 0 | 0 | 0 | 1 | 2 |
| MIMAT0002842 | hsa-miR-518f-3p | MTHFSD | 64779 | 0 | 2 | 0 | 0 | 0 | 1 | 2 |
| MIMAT0002842 | hsa-miR-518f-3p | NFATC3 | 4775 | 0 | 1 | 0 | 0 | 0 | 1 | 2 |
| MIMAT0002842 | hsa-miR-518f-3p | OTP | 23440 | 0 | 2 | 0 | 0 | 0 | 1 | 2 |
| MIMAT0002842 | hsa-miR-518f-3p | PDE2A | 5138 | 0 | 1 | 0 | 0 | 0 | 1 | 2 |
| MIMAT0002842 | hsa-miR-518f-3p | RBBP8 | 5932 | 0 | 1 | 0 | 0 | 0 | 1 | 2 |
| MIMAT0002842 | hsa-miR-518f-3p | RGCC | 28984 | 0 | 1 | 0 | 0 | 0 | 1 | 2 |
| MIMAT0002842 | hsa-miR-518f-3p | SLC4A2 | 6522 | 0 | 1 | 0 | 0 | 0 | 1 | 2 |
| MIMAT0002842 | hsa-miR-518f-3p | SPATS2L | 26010 | 0 | 1 | 0 | 0 | 0 | 1 | 2 |
| MIMAT0002842 | hsa-miR-518f-3p | SUGT1 | 10910 | 0 | 0 | 1 | 0 | 0 | 1 | 2 |
| MIMAT0002842 | hsa-miR-518f-3p | TSN | 7247 | 0 | 0 | 2 | 2 | 0 | 0 | 2 |
| MIMAT0002842 | hsa-miR-518f-3p | YLPM1 | 56252 | 0 | 1 | 0 | 0 | 0 | 1 | 2 |
| MIMAT0002842 | hsa-miR-518f-3p | ZNF282 | 8427 | 1 | 0 | 2 | 0 | 0 | 0 | 2 |
| MIMAT0003265 | hsa-miR-597-5p | ARHGEF4 | 50649 | 1 | 0 | 2 | 0 | 0 | 0 | 2 |
| MIMAT0003265 | hsa-miR-597-5p | ATP2A1 | 487 | 0 | 1 | 1 | 0 | 0 | 0 | 2 |
| MIMAT0003265 | hsa-miR-597-5p | BCCIP | 56647 | 1 | 0 | 0 | 1 | 0 | 0 | 2 |
| MIMAT0003265 | hsa-miR-597-5p | BOD1L1 | 259282 | 0 | 0 | 1 | 1 | 0 | 0 | 2 |
| MIMAT0003265 | hsa-miR-597-5p | BRIP1 | 83990 | 0 | 1 | 0 | 1 | 0 | 0 | 2 |
| MIMAT0003265 | hsa-miR-597-5p | CCDC178 | 374864 | 0 | 0 | 1 | 0 | 0 | 1 | 2 |
| MIMAT0003265 | hsa-miR-597-5p | CCDC50 | 152137 | 0 | 1 | 0 | 0 | 0 | 1 | 2 |
| MIMAT0003265 | hsa-miR-597-5p | DCAF12 | 25853 | 1 | 0 | 0 | 0 | 0 | 1 | 2 |
| MIMAT0003265 | hsa-miR-597-5p | DPPA2 | 151871 | 0 | 1 | 1 | 0 | 0 | 0 | 2 |
| MIMAT0003265 | hsa-miR-597-5p | FAM126B | 285172 | 1 | 0 | 0 | 0 | 0 | 1 | 2 |
| MIMAT0003265 | hsa-miR-597-5p | FGF14 | 2259 | 0 | 0 | 1 | 0 | 1 | 0 | 2 |
| MIMAT0003265 | hsa-miR-597-5p | FGFR1OP2 | 26127 | 1 | 0 | 0 | 1 | 0 | 0 | 2 |
| MIMAT0003265 | hsa-miR-597-5p | FOXO3 | 2309 | 1 | 0 | 0 | 0 | 0 | 2 | 2 |
| MIMAT0003265 | hsa-miR-597-5p | GABRA2 | 2555 | 1 | 1 | 0 | 0 | 0 | 0 | 2 |
| MIMAT0003265 | hsa-miR-597-5p | GJB7 | 375519 | 1 | 0 | 0 | 1 | 0 | 0 | 2 |
| MIMAT0003265 | hsa-miR-597-5p | GPR158 | 57512 | 1 | 0 | 1 | 0 | 0 | 0 | 2 |
| MIMAT0003265 | hsa-miR-597-5p | HDC | 3067 | 0 | 1 | 0 | 0 | 0 | 1 | 2 |
| MIMAT0003265 | hsa-miR-597-5p | ING3 | 54556 | 1 | 0 | 0 | 0 | 0 | 1 | 2 |
| MIMAT0003265 | hsa-miR-597-5p | ITGB1BP1 | 9270 | 0 | 1 | 0 | 0 | 0 | 1 | 2 |
| MIMAT0003265 | hsa-miR-597-5p | LAMC1 | 3915 | 0 | 0 | 0 | 0 | 1 | 1 | 2 |
| MIMAT0003265 | hsa-miR-597-5p | LCORL | 254251 | 1 | 0 | 0 | 0 | 1 | 0 | 2 |
| MIMAT0003265 | hsa-miR-597-5p | LRRC4C | 57689 | 1 | 0 | 0 | 0 | 0 | 1 | 2 |
| MIMAT0003265 | hsa-miR-597-5p | MAFG | 4097 | 0 | 0 | 0 | 0 | 1 | 1 | 2 |
| MIMAT0003265 | hsa-miR-597-5p | MICALCL | 84953 | 1 | 0 | 1 | 0 | 0 | 0 | 2 |
| MIMAT0003265 | hsa-miR-597-5p | NCAM1 | 4684 | 1 | 0 | 0 | 0 | 0 | 2 | 2 |
| MIMAT0003265 | hsa-miR-597-5p | NRK | 203447 | 0 | 0 | 1 | 1 | 0 | 0 | 2 |
| MIMAT0003265 | hsa-miR-597-5p | OBSL1 | 23363 | 0 | 1 | 0 | 1 | 0 | 0 | 2 |
| MIMAT0003265 | hsa-miR-597-5p | OLFML1 | 283298 | 0 | 0 | 1 | 0 | 0 | 1 | 2 |
| MIMAT0003265 | hsa-miR-597-5p | OPA3 | 80207 | 1 | 0 | 0 | 1 | 0 | 0 | 2 |
| MIMAT0003265 | hsa-miR-597-5p | OSM | 5008 | 0 | 0 | 0 | 0 | 1 | 1 | 2 |
| MIMAT0003265 | hsa-miR-597-5p | PAFAH1B1 | 5048 | 1 | 0 | 0 | 0 | 0 | 2 | 2 |
| MIMAT0003265 | hsa-miR-597-5p | PARP2 | 10038 | 1 | 0 | 1 | 0 | 0 | 0 | 2 |
| MIMAT0003265 | hsa-miR-597-5p | PGRMC1 | 10857 | 1 | 0 | 1 | 0 | 0 | 0 | 2 |
| MIMAT0003265 | hsa-miR-597-5p | PHKA2 | 5256 | 1 | 0 | 0 | 0 | 0 | 1 | 2 |
| MIMAT0003265 | hsa-miR-597-5p | PRKX | 5613 | 1 | 0 | 0 | 1 | 0 | 0 | 2 |
| MIMAT0003265 | hsa-miR-597-5p | PRRC2B | 84726 | 0 | 2 | 0 | 0 | 0 | 1 | 2 |
| MIMAT0003265 | hsa-miR-597-5p | PTBP1 | 100616459 | 1 | 0 | 0 | 0 | 0 | 1 | 2 |
| MIMAT0003265 | hsa-miR-597-5p | PTBP1 | 5725 | 1 | 0 | 0 | 0 | 0 | 1 | 2 |
| MIMAT0003265 | hsa-miR-597-5p | PWWP2A | 114825 | 1 | 0 | 0 | 1 | 0 | 0 | 2 |
| MIMAT0003265 | hsa-miR-597-5p | RUNX2 | 860 | 0 | 0 | 1 | 0 | 0 | 1 | 2 |
| MIMAT0003265 | hsa-miR-597-5p | SCN2A | 6326 | 0 | 0 | 1 | 2 | 0 | 0 | 2 |
| MIMAT0003265 | hsa-miR-597-5p | SGK1 | 6446 | 0 | 0 | 1 | 0 | 0 | 1 | 2 |
| MIMAT0003265 | hsa-miR-597-5p | SLC18A2 | 6571 | 1 | 0 | 1 | 0 | 0 | 0 | 2 |
| MIMAT0003265 | hsa-miR-597-5p | SLC31A1 | 1317 | 0 | 1 | 0 | 0 | 0 | 1 | 2 |
| MIMAT0003265 | hsa-miR-597-5p | SPRY1 | 10252 | 0 | 0 | 1 | 0 | 0 | 1 | 2 |
| MIMAT0003265 | hsa-miR-597-5p | SRSF3 | 6428 | 1 | 0 | 1 | 0 | 0 | 0 | 2 |
| MIMAT0003265 | hsa-miR-597-5p | TAF15 | 8148 | 0 | 1 | 0 | 0 | 0 | 1 | 2 |
| MIMAT0003265 | hsa-miR-597-5p | TBC1D31 | 93594 | 1 | 0 | 2 | 0 | 0 | 0 | 2 |
| MIMAT0003265 | hsa-miR-597-5p | TLX1 | 3195 | 0 | 1 | 0 | 2 | 0 | 0 | 2 |
| MIMAT0003265 | hsa-miR-597-5p | TMEM87A | 25963 | 1 | 0 | 1 | 0 | 0 | 0 | 2 |
| MIMAT0003265 | hsa-miR-597-5p | TRAPPC3 | 27095 | 1 | 1 | 0 | 0 | 0 | 0 | 2 |
| MIMAT0003265 | hsa-miR-597-5p | TRIQK | 286144 | 1 | 0 | 1 | 0 | 0 | 0 | 2 |
| MIMAT0003265 | hsa-miR-597-5p | TTI1 | 9675 | 0 | 1 | 0 | 0 | 0 | 1 | 2 |
| MIMAT0003265 | hsa-miR-597-5p | UBE4B | 10277 | 0 | 0 | 1 | 0 | 0 | 1 | 2 |
| MIMAT0003265 | hsa-miR-597-5p | USP51 | 158880 | 1 | 1 | 0 | 0 | 0 | 0 | 2 |
| MIMAT0003265 | hsa-miR-597-5p | ZC3H12C | 85463 | 0 | 0 | 0 | 1 | 1 | 0 | 2 |
| MIMAT0003265 | hsa-miR-597-5p | ZMYND11 | 10771 | 1 | 0 | 0 | 0 | 0 | 1 | 2 |
| MIMAT0005911 | hsa-miR-1260a | ACVR1B | 91 | 0 | 0 | 0 | 0 | 1 | 1 | 2 |
| MIMAT0005911 | hsa-miR-1260a | ADAM11 | 4185 | 0 | 0 | 0 | 0 | 1 | 1 | 2 |
| MIMAT0005911 | hsa-miR-1260a | ADIPOR2 | 79602 | 1 | 0 | 0 | 0 | 1 | 0 | 2 |
| MIMAT0005911 | hsa-miR-1260a | ANKRD34A | 101060487 | 1 | 0 | 0 | 0 | 1 | 0 | 2 |
| MIMAT0005911 | hsa-miR-1260a | ANKRD34A | 284615 | 1 | 0 | 0 | 0 | 1 | 0 | 2 |
| MIMAT0005911 | hsa-miR-1260a | ANKRD52 | 283373 | 0 | 0 | 0 | 0 | 1 | 2 | 2 |
| MIMAT0005911 | hsa-miR-1260a | ANTXR2 | 118429 | 0 | 0 | 0 | 0 | 1 | 1 | 2 |
| MIMAT0005911 | hsa-miR-1260a | ARL3 | 403 | 0 | 0 | 0 | 0 | 1 | 1 | 2 |
| MIMAT0005911 | hsa-miR-1260a | ARPP21 | 10777 | 0 | 0 | 1 | 0 | 1 | 0 | 2 |
| MIMAT0005911 | hsa-miR-1260a | ASH1L | 55870 | 0 | 0 | 0 | 0 | 1 | 1 | 2 |
| MIMAT0005911 | hsa-miR-1260a | ATP2B1 | 490 | 0 | 0 | 0 | 0 | 2 | 1 | 2 |
| MIMAT0005911 | hsa-miR-1260a | BASP1 | 10409 | 0 | 0 | 0 | 0 | 1 | 1 | 2 |
| MIMAT0005911 | hsa-miR-1260a | BCL2L2 | 599 | 1 | 0 | 0 | 0 | 1 | 0 | 2 |
| MIMAT0005911 | hsa-miR-1260a | BCL9L | 283149 | 0 | 0 | 0 | 0 | 1 | 1 | 2 |
| MIMAT0005911 | hsa-miR-1260a | BRK1 | 55845 | 0 | 0 | 0 | 1 | 0 | 1 | 2 |
| MIMAT0005911 | hsa-miR-1260a | BRSK1 | 84446 | 0 | 0 | 0 | 0 | 1 | 1 | 2 |
| MIMAT0005911 | hsa-miR-1260a | C14orf1 | 11161 | 1 | 0 | 0 | 0 | 1 | 0 | 2 |
| MIMAT0005911 | hsa-miR-1260a | C17orf64 | 124773 | 1 | 0 | 1 | 0 | 0 | 0 | 2 |
| MIMAT0005911 | hsa-miR-1260a | C17orf85 | 55421 | 0 | 0 | 0 | 0 | 1 | 1 | 2 |
| MIMAT0005911 | hsa-miR-1260a | C19orf44 | 84167 | 0 | 0 | 0 | 0 | 1 | 1 | 2 |
| MIMAT0005911 | hsa-miR-1260a | C1orf63 | 57035 | 1 | 0 | 0 | 0 | 0 | 1 | 2 |
| MIMAT0005911 | hsa-miR-1260a | C20orf112 | 140688 | 0 | 0 | 0 | 0 | 1 | 1 | 2 |
| MIMAT0005911 | hsa-miR-1260a | C6orf132 | 647024 | 1 | 0 | 0 | 0 | 1 | 0 | 2 |
| MIMAT0005911 | hsa-miR-1260a | C7orf60 | 154743 | 0 | 0 | 0 | 0 | 1 | 2 | 2 |
| MIMAT0005911 | hsa-miR-1260a | C9orf40 | 55071 | 1 | 0 | 1 | 0 | 0 | 0 | 2 |
| MIMAT0005911 | hsa-miR-1260a | CAMKV | 79012 | 0 | 0 | 0 | 0 | 1 | 1 | 2 |
| MIMAT0005911 | hsa-miR-1260a | CDC42 | 998 | 0 | 0 | 0 | 0 | 1 | 1 | 2 |
| MIMAT0005911 | hsa-miR-1260a | CDK16 | 5127 | 0 | 0 | 0 | 0 | 1 | 1 | 2 |
| MIMAT0005911 | hsa-miR-1260a | CLRN1 | 7401 | 0 | 0 | 1 | 2 | 0 | 0 | 2 |
| MIMAT0005911 | hsa-miR-1260a | CSNK2A1 | 1457 | 0 | 0 | 0 | 0 | 1 | 1 | 2 |
| MIMAT0005911 | hsa-miR-1260a | CSNK2B | 1460 | 0 | 0 | 4 | 1 | 0 | 0 | 2 |
| MIMAT0005911 | hsa-miR-1260a | CSNK2B | 1460 | 0 | 0 | 4 | 1 | 0 | 0 | 2 |
| MIMAT0005911 | hsa-miR-1260a | CSNK2B | 1460 | 0 | 0 | 4 | 1 | 0 | 0 | 2 |
| MIMAT0005911 | hsa-miR-1260a | CSNK2B | 1460 | 0 | 0 | 4 | 1 | 0 | 0 | 2 |
| MIMAT0005911 | hsa-miR-1260a | CSNK2B | 1460 | 0 | 0 | 4 | 1 | 0 | 0 | 2 |
| MIMAT0005911 | hsa-miR-1260a | CSNK2B | 1460 | 0 | 0 | 4 | 1 | 0 | 0 | 2 |
| MIMAT0005911 | hsa-miR-1260a | CSNK2B | 1460 | 0 | 0 | 4 | 1 | 0 | 0 | 2 |
| MIMAT0005911 | hsa-miR-1260a | CTAGE1 | 64693 | 1 | 0 | 0 | 1 | 0 | 0 | 2 |
| MIMAT0005911 | hsa-miR-1260a | CUL2 | 8453 | 0 | 0 | 0 | 0 | 1 | 1 | 2 |
| MIMAT0005911 | hsa-miR-1260a | CXorf23 | 256643 | 1 | 0 | 0 | 0 | 1 | 0 | 2 |
| MIMAT0005911 | hsa-miR-1260a | DDX27 | 55661 | 0 | 0 | 0 | 0 | 1 | 1 | 2 |
| MIMAT0005911 | hsa-miR-1260a | DES | 1674 | 0 | 0 | 0 | 0 | 1 | 1 | 2 |
| MIMAT0005911 | hsa-miR-1260a | DLG4 | 1742 | 0 | 0 | 0 | 0 | 1 | 1 | 2 |
| MIMAT0005911 | hsa-miR-1260a | DNAJC14 | 85406 | 0 | 0 | 0 | 0 | 1 | 1 | 2 |
| MIMAT0005911 | hsa-miR-1260a | EIF4E3 | 317649 | 0 | 0 | 0 | 0 | 1 | 1 | 2 |
| MIMAT0005911 | hsa-miR-1260a | ELAVL3 | 1995 | 0 | 0 | 0 | 0 | 1 | 1 | 2 |
| MIMAT0005911 | hsa-miR-1260a | ELK1 | 2002 | 0 | 0 | 0 | 0 | 2 | 2 | 2 |
| MIMAT0005911 | hsa-miR-1260a | ESRRA | 2101 | 0 | 0 | 0 | 0 | 1 | 1 | 2 |
| MIMAT0005911 | hsa-miR-1260a | ETS1 | 2113 | 0 | 0 | 0 | 0 | 1 | 1 | 2 |
| MIMAT0005911 | hsa-miR-1260a | ETV3 | 2117 | 1 | 0 | 0 | 0 | 1 | 0 | 2 |
| MIMAT0005911 | hsa-miR-1260a | FAM131B | 9715 | 0 | 0 | 0 | 0 | 1 | 1 | 2 |
| MIMAT0005911 | hsa-miR-1260a | FAM53C | 51307 | 0 | 0 | 0 | 0 | 1 | 1 | 2 |
| MIMAT0005911 | hsa-miR-1260a | FBXL19 | 54620 | 0 | 0 | 0 | 0 | 1 | 2 | 2 |
| MIMAT0005911 | hsa-miR-1260a | FRAT2 | 23401 | 1 | 0 | 0 | 0 | 1 | 0 | 2 |
| MIMAT0005911 | hsa-miR-1260a | GABRA4 | 2557 | 0 | 0 | 0 | 0 | 1 | 1 | 2 |
| MIMAT0005911 | hsa-miR-1260a | GATAD2B | 57459 | 0 | 0 | 0 | 0 | 1 | 3 | 2 |
| MIMAT0005911 | hsa-miR-1260a | GDF11 | 10220 | 0 | 0 | 0 | 0 | 1 | 2 | 2 |
| MIMAT0005911 | hsa-miR-1260a | GGT7 | 2686 | 0 | 0 | 0 | 0 | 1 | 1 | 2 |
| MIMAT0005911 | hsa-miR-1260a | HAS3 | 3038 | 0 | 0 | 0 | 0 | 1 | 1 | 2 |
| MIMAT0005911 | hsa-miR-1260a | HNRNPUL2 | 221092 | 0 | 0 | 0 | 0 | 1 | 1 | 2 |
| MIMAT0005911 | hsa-miR-1260a | IRX1 | 79192 | 0 | 0 | 0 | 0 | 1 | 1 | 2 |
| MIMAT0005911 | hsa-miR-1260a | JPH4 | 84502 | 1 | 0 | 0 | 0 | 1 | 0 | 2 |
| MIMAT0005911 | hsa-miR-1260a | KDM3B | 51780 | 0 | 0 | 1 | 0 | 0 | 1 | 2 |
| MIMAT0005911 | hsa-miR-1260a | KIAA2022 | 340533 | 0 | 0 | 0 | 0 | 1 | 2 | 2 |
| MIMAT0005911 | hsa-miR-1260a | KIF21A | 55605 | 0 | 0 | 1 | 0 | 1 | 0 | 2 |
| MIMAT0005911 | hsa-miR-1260a | KIF26A | 26153 | 0 | 0 | 0 | 0 | 1 | 1 | 2 |
| MIMAT0005911 | hsa-miR-1260a | KMT2D | 8085 | 0 | 0 | 0 | 0 | 1 | 1 | 2 |
| MIMAT0005911 | hsa-miR-1260a | KPNA6 | 23633 | 0 | 0 | 0 | 0 | 1 | 2 | 2 |
| MIMAT0005911 | hsa-miR-1260a | LHX1 | 3975 | 1 | 0 | 0 | 0 | 1 | 0 | 2 |
| MIMAT0005911 | hsa-miR-1260a | LIMK1 | 3984 | 0 | 0 | 0 | 0 | 1 | 1 | 2 |
| MIMAT0005911 | hsa-miR-1260a | LRRTM3 | 347731 | 1 | 0 | 0 | 0 | 1 | 0 | 2 |
| MIMAT0005911 | hsa-miR-1260a | MAPT | 4137 | 0 | 0 | 0 | 0 | 1 | 1 | 2 |
| MIMAT0005911 | hsa-miR-1260a | MARCH7 | 64844 | 0 | 0 | 0 | 0 | 1 | 1 | 2 |
| MIMAT0005911 | hsa-miR-1260a | MECP2 | 4204 | 0 | 0 | 0 | 0 | 1 | 1 | 2 |
| MIMAT0005911 | hsa-miR-1260a | MED1 | 5469 | 0 | 0 | 0 | 0 | 1 | 1 | 2 |
| MIMAT0005911 | hsa-miR-1260a | MEX3A | 92312 | 0 | 0 | 0 | 0 | 1 | 1 | 2 |
| MIMAT0005911 | hsa-miR-1260a | MSL2 | 55167 | 0 | 0 | 0 | 0 | 1 | 2 | 2 |
| MIMAT0005911 | hsa-miR-1260a | MSTO1 | 55154 | 0 | 0 | 3 | 0 | 0 | 1 | 2 |
| MIMAT0005911 | hsa-miR-1260a | MYT1L | 23040 | 0 | 0 | 0 | 0 | 1 | 1 | 2 |
| MIMAT0005911 | hsa-miR-1260a | NAV1 | 89796 | 0 | 0 | 0 | 0 | 1 | 1 | 2 |
| MIMAT0005911 | hsa-miR-1260a | NFIB | 4781 | 0 | 0 | 0 | 0 | 1 | 1 | 2 |
| MIMAT0005911 | hsa-miR-1260a | NHS | 4810 | 0 | 0 | 0 | 0 | 1 | 1 | 2 |
| MIMAT0005911 | hsa-miR-1260a | NPAS4 | 266743 | 0 | 0 | 0 | 0 | 1 | 1 | 2 |
| MIMAT0005911 | hsa-miR-1260a | NRXN2 | 9379 | 0 | 0 | 0 | 0 | 1 | 2 | 2 |
| MIMAT0005911 | hsa-miR-1260a | NXF1 | 10482 | 0 | 0 | 0 | 0 | 1 | 1 | 2 |
| MIMAT0005911 | hsa-miR-1260a | OTX1 | 5013 | 1 | 0 | 0 | 0 | 1 | 0 | 2 |
| MIMAT0005911 | hsa-miR-1260a | PARM1 | 25849 | 1 | 0 | 0 | 0 | 0 | 1 | 2 |
| MIMAT0005911 | hsa-miR-1260a | PCDH19 | 57526 | 0 | 0 | 0 | 0 | 1 | 1 | 2 |
| MIMAT0005911 | hsa-miR-1260a | PDIA3 | 2923 | 0 | 0 | 0 | 0 | 1 | 1 | 2 |
| MIMAT0005911 | hsa-miR-1260a | PHF21A | 51317 | 0 | 0 | 0 | 0 | 1 | 1 | 2 |
| MIMAT0005911 | hsa-miR-1260a | PI4K2A | 55361 | 0 | 0 | 0 | 0 | 1 | 1 | 2 |
| MIMAT0005911 | hsa-miR-1260a | PIP5K1A | 8394 | 0 | 0 | 0 | 0 | 1 | 1 | 2 |
| MIMAT0005911 | hsa-miR-1260a | POU3F1 | 5453 | 0 | 0 | 0 | 0 | 1 | 1 | 2 |
| MIMAT0005911 | hsa-miR-1260a | PPP1R8 | 5511 | 0 | 0 | 0 | 0 | 1 | 2 | 2 |
| MIMAT0005911 | hsa-miR-1260a | PSD | 5662 | 0 | 0 | 0 | 0 | 1 | 1 | 2 |
| MIMAT0005911 | hsa-miR-1260a | PTGFRN | 5738 | 1 | 0 | 0 | 0 | 1 | 0 | 2 |
| MIMAT0005911 | hsa-miR-1260a | PTPRD | 5789 | 0 | 0 | 0 | 0 | 1 | 1 | 2 |
| MIMAT0005911 | hsa-miR-1260a | PTPRK | 5796 | 0 | 0 | 0 | 0 | 1 | 1 | 2 |
| MIMAT0005911 | hsa-miR-1260a | RAB6B | 51560 | 0 | 0 | 0 | 0 | 1 | 1 | 2 |
| MIMAT0005911 | hsa-miR-1260a | RAD23B | 5887 | 0 | 0 | 0 | 0 | 1 | 1 | 2 |
| MIMAT0005911 | hsa-miR-1260a | RLF | 6018 | 0 | 0 | 0 | 0 | 1 | 1 | 2 |
| MIMAT0005911 | hsa-miR-1260a | RNF41 | 10193 | 1 | 0 | 0 | 0 | 1 | 0 | 2 |
| MIMAT0005911 | hsa-miR-1260a | RYBP | 23429 | 0 | 0 | 0 | 0 | 1 | 1 | 2 |
| MIMAT0005911 | hsa-miR-1260a | SCD | 6319 | 1 | 0 | 0 | 0 | 1 | 0 | 2 |
| MIMAT0005911 | hsa-miR-1260a | SEMA3A | 10371 | 0 | 0 | 1 | 1 | 0 | 0 | 2 |
| MIMAT0005911 | hsa-miR-1260a | SLC16A3 | 9123 | 1 | 0 | 0 | 0 | 1 | 0 | 2 |
| MIMAT0005911 | hsa-miR-1260a | SLC20A1 | 6574 | 0 | 0 | 0 | 0 | 1 | 2 | 2 |
| MIMAT0005911 | hsa-miR-1260a | SMAP2 | 64744 | 0 | 0 | 0 | 1 | 1 | 0 | 2 |
| MIMAT0005911 | hsa-miR-1260a | SMARCD1 | 6602 | 0 | 0 | 0 | 0 | 1 | 1 | 2 |
| MIMAT0005911 | hsa-miR-1260a | SPEN | 23013 | 0 | 0 | 1 | 1 | 0 | 0 | 2 |
| MIMAT0005911 | hsa-miR-1260a | SPRY2 | 10253 | 0 | 0 | 1 | 0 | 0 | 1 | 2 |
| MIMAT0005911 | hsa-miR-1260a | SRF | 6722 | 0 | 0 | 0 | 0 | 1 | 1 | 2 |
| MIMAT0005911 | hsa-miR-1260a | SRSF2 | 6427 | 0 | 0 | 0 | 0 | 1 | 1 | 2 |
| MIMAT0005911 | hsa-miR-1260a | SRSF2 | 693221 | 0 | 0 | 0 | 0 | 1 | 1 | 2 |
| MIMAT0005911 | hsa-miR-1260a | STAG2 | 10735 | 0 | 0 | 0 | 0 | 1 | 1 | 2 |
| MIMAT0005911 | hsa-miR-1260a | STMN4 | 81551 | 0 | 0 | 0 | 0 | 1 | 1 | 2 |
| MIMAT0005911 | hsa-miR-1260a | TAOK3 | 51347 | 0 | 0 | 0 | 0 | 1 | 1 | 2 |
| MIMAT0005911 | hsa-miR-1260a | TCF20 | 6942 | 0 | 0 | 0 | 0 | 2 | 2 | 2 |
| MIMAT0005911 | hsa-miR-1260a | TIMM17A | 10440 | 1 | 0 | 0 | 0 | 1 | 0 | 2 |
| MIMAT0005911 | hsa-miR-1260a | TMEM104 | 54868 | 1 | 0 | 0 | 0 | 1 | 0 | 2 |
| MIMAT0005911 | hsa-miR-1260a | TMEM178B | 100507421 | 0 | 0 | 0 | 1 | 1 | 0 | 2 |
| MIMAT0005911 | hsa-miR-1260a | TNPO1 | 3842 | 0 | 0 | 0 | 0 | 1 | 1 | 2 |
| MIMAT0005911 | hsa-miR-1260a | TOP1 | 7150 | 0 | 0 | 1 | 0 | 1 | 0 | 2 |
| MIMAT0005911 | hsa-miR-1260a | TSC22D2 | 9819 | 0 | 0 | 1 | 1 | 0 | 0 | 2 |
| MIMAT0005911 | hsa-miR-1260a | TTC14 | 151613 | 0 | 0 | 0 | 0 | 1 | 1 | 2 |
| MIMAT0005911 | hsa-miR-1260a | UBE2E1 | 7324 | 0 | 0 | 0 | 0 | 1 | 1 | 2 |
| MIMAT0005911 | hsa-miR-1260a | UBTF | 7343 | 1 | 0 | 0 | 0 | 1 | 0 | 2 |
| MIMAT0005911 | hsa-miR-1260a | UNC13C | 440279 | 0 | 0 | 1 | 0 | 1 | 0 | 2 |
| MIMAT0005911 | hsa-miR-1260a | VAMP1 | 6843 | 0 | 0 | 0 | 0 | 1 | 1 | 2 |
| MIMAT0005911 | hsa-miR-1260a | VIPAS39 | 63894 | 1 | 0 | 0 | 0 | 1 | 0 | 2 |
| MIMAT0005911 | hsa-miR-1260a | VPS4A | 27183 | 1 | 0 | 0 | 0 | 0 | 1 | 2 |
| MIMAT0005911 | hsa-miR-1260a | WBP11 | 51729 | 0 | 0 | 0 | 0 | 1 | 1 | 2 |
| MIMAT0005911 | hsa-miR-1260a | WDFY3 | 23001 | 0 | 0 | 0 | 0 | 1 | 1 | 2 |
| MIMAT0005911 | hsa-miR-1260a | WIBG | 84305 | 0 | 0 | 1 | 0 | 0 | 1 | 2 |
| MIMAT0005911 | hsa-miR-1260a | XPO7 | 23039 | 0 | 0 | 0 | 0 | 1 | 2 | 2 |
| MIMAT0005911 | hsa-miR-1260a | XPR1 | 9213 | 1 | 0 | 0 | 0 | 1 | 0 | 2 |
| MIMAT0005911 | hsa-miR-1260a | ZBTB6 | 10773 | 1 | 0 | 0 | 0 | 0 | 1 | 2 |
| MIMAT0005911 | hsa-miR-1260a | ZFP90 | 146198 | 1 | 0 | 0 | 1 | 0 | 0 | 2 |
| MIMAT0005911 | hsa-miR-1260a | ZFYVE9 | 9372 | 0 | 0 | 0 | 1 | 1 | 0 | 2 |
| MIMAT0005911 | hsa-miR-1260a | ZNF219 | 51222 | 0 | 0 | 0 | 0 | 1 | 2 | 2 |
| MIMAT0005911 | hsa-miR-1260a | ZNF367 | 195828 | 0 | 0 | 0 | 1 | 1 | 0 | 2 |
| MIMAT0005911 | hsa-miR-1260a | ZNF384 | 171017 | 0 | 0 | 0 | 1 | 1 | 0 | 2 |
| MIMAT0005911 | hsa-miR-1260a | ZNF48 | 197407 | 1 | 0 | 0 | 0 | 1 | 0 | 2 |
| MIMAT0005911 | hsa-miR-1260a | ZNF763 | 284390 | 0 | 0 | 0 | 1 | 0 | 1 | 2 |
| MIMAT0005911 | hsa-miR-1260a | ZNF781 | 163115 | 1 | 0 | 0 | 1 | 0 | 0 | 2 |
| MIMAT0005911 | hsa-miR-1260a | ZNF814 | 730051 | 1 | 0 | 0 | 0 | 1 | 0 | 2 |

**Supplementary Table 4**. Gene Ontology analysis was performed with the predicted targets of the miRNA miR-1260a, miR-518f-3p and miR-597-5p using STRING database. For the enrichment analysis, the genome was used as statistical background. Only terms with a FDR<0.00001 are reported.

| **#term ID** | **term description** | **observed gene count** | **background gene count** | **strength** | **FDR** |
| --- | --- | --- | --- | --- | --- |
| **GO:0010468** | regulation of gene expression | 101 | 4533 | 0.23 | 1.29e-05 |
| **GO:0051171** | regulation of nitrogen compound metabolic process | 118 | 5827 | 0.19 | 2.11e-05 |
| **GO:0051252** | regulation of RNA metabolic process | 89 | 3890 | 0.25 | 2.11e-05 |
| **GO:0080090** | regulation of primary metabolic process | 121 | 5982 | 0.19 | 2.11e-05 |
| **GO:0019219** | regulation of nucleobase-containing compound metabolic process | 92 | 4133 | 0.23 | 2.13e-05 |
| **GO:0019222** | regulation of metabolic process | 127 | 6516 | 0.18 | 2.13e-05 |
| **GO:0060255** | regulation of macromolecule metabolic process | 121 | 6072 | 0.19 | 2.13e-05 |
| **GO:0009889** | regulation of biosynthetic process | 94 | 4337 | 0.22 | 2.97e-05 |
| **GO:0031323** | regulation of cellular metabolic process | 120 | 6082 | 0.18 | 2.97e-05 |
| **GO:2000112** | regulation of cellular macromolecule biosynthetic process | 89 | 4050 | 0.23 | 3.94e-05 |
| **GO:0007399** | nervous system development | 58 | 2206 | 0.31 | 4.77e-05 |
| **GO:0010556** | regulation of macromolecule biosynthetic process | 90 | 4143 | 0.22 | 4.77e-05 |
| **GO:0032502** | developmental process | 109 | 5401 | 0.19 | 4.77e-05 |
| **GO:0006357** | regulation of transcription by RNA polymerase II | 65 | 2633 | 0.28 | 5.89e-05 |
| **GO:0007275** | multicellular organism development | 98 | 4726 | 0.2 | 6.71e-05 |
| **GO:0031326** | regulation of cellular biosynthetic process | 91 | 4266 | 0.22 | 6.71e-05 |
| **GO:0090304** | nucleic acid metabolic process | 86 | 3941 | 0.23 | 6.71e-05 |
| **GO:0048856** | anatomical structure development | 103 | 5085 | 0.19 | 7.27e-05 |

**Supplementary Data 1. Script of the multiMiR R package used for miRNAs’ targets prediction.**

#### load package library(multiMiR)

###parse

##sel mirna

list_mirna<- c("hsa-miR-1260a", "hsa-miR-597-5p", "hsa-miR-518f-3p")

##get analysis list_targets<-multiMiR::get_multimir(org="hsa", mirna=list_mirna,

table = "predicted", predicted.cutoff.type = "n", summary=TRUE)

##define targets with multiple detect

groups_list <- rep(0, dim(list_targets@summary)[1])

groups_list[list_targets@summary$predicted.sum >="2"] <- 1

list_targets_summary_selected <- list_targets@summary[which(groups_list!=0),]

###generate table

list_targets_summary<- as.data.frame(list_targets@summary)

list_targets_summary_selected <- as.data.frame(list_targets@summary[which(groups_list!=0),])

###export

write.table(list_targets_summary,"list_targets_summary.txt", sep="\t",row.names = F)

write.table(list_targets_summary_selected,"list_targets_summary_selected.txt", sep="\t",row.names = F)

1. miRNA with a *p* value (unadjusted) < 0.05 are highlighted in grey [↑](#footnote-ref-1)
